# Synthesis and In Vitro Antiproliferative Activity of Novel Phenyl Ring-Substituted 5-Alkyl-12(*H*)-quino[3,4-*b*][1,4]benzothiazine Derivatives

**DOI:** 10.3390/molecules21111455

**Published:** 2016-11-04

**Authors:** Andrzej Zięba, Małgorzata Latocha, Aleksander Sochanik, Anna Nycz, Dariusz Kuśmierz

**Affiliations:** 1 Department of Organic Chemistry, School of Pharmacy with the Division of Laboratory Medicine in Sosnowiec, Medical University of Silesia in Katowice, Jagiellońska 4, 41-200 Sosnowiec, Poland; anna.nycz00@gmail.com; 2 Department of Cell Biology, School of Pharmacy with the Division of Laboratory Medicine in Sosnowiec, Medical University of Silesia in Katowice, Jedności 9, 41-200 Sosnowiec, Poland; mlatocha@sum.edu.pl (M.L.); kusmierz@sum.edu.pl (D.K.); 3 Center for Translational Research and Molecular Biology of Cancer, Maria Skłodowska-Curie Memorial Cancer Center and Institute of Oncology, Wybrzeże AK 15, 44-101 Gliwice, Poland; aleksander.sochanik@io.gliwice.pl

**Keywords:** phenothiazine, azaphenothiazine, anticancer, cisplatin

## Abstract

A novel series of tetracyclic quinobenzothiazine derivatives was synthetized. Compounds containing a substituent (hydroxyl, methyl, phenyl, piperidyl, or piperazinyl) in positions 9 and 11 were obtained by cyclization of suitable 4-aminoquinolinium-3-thiolates. Quinobenzothiazine 10-*O*-substituted derivatives were obtained by alkylating the hydroxyl group in position 10 of the parent (quinobenzothiazine) system. Antiproliferative activity of the synthesized compounds was studied using cultured neoplastic cells (MDA-MB-231, SNB-19, and C-32 cell lines). Four selected compounds were investigated in more detail for cytotoxicity and antiproliferative effect. Transcriptional activity of genes regulating cell cycle (TP53), apoptosis (BAX, BCL-2), as well as proliferation (H3) were assessed. Finally, the ability of the selected compounds to bind DNA was checked in the presence of ethidium bromide.

## 1. Introduction

Initial attempts of using phenothiazine derivatives as antimalarial agents go back to 1891 when Guttman and Ehrlich demonstrated chemotherapeutic effectiveness of methylene blue. Extensive search for antimalarial agents was undertaken later, starting with modification of the methylene blue structure via substitution of the N-methyl group with alkylaminoalkyl moieties [1]. Studies concerning antihistamine activity led to phenothiazine derivatives that contain alkylaminoalkyl substituents at the thiazine nitrogen atom. Compounds containing such a structural fragment are important neuroleptic agents. Their main representative, and a reference compound, is chlorpromazine [2]. Phenothiazines are typical examples of how modification of a basic structural fragment can affect directional activity and drug strength. Search for new therapeutics has been conducted more and more often by modifying the main structural fragment of known drugs. Since the advent of chlorpromazine, the quest for new phenothiazine derivatives has yielded several thousand compounds with novel properties and applications. Exchanging benzene rings for nitrogen-containing heterocycles has led to a series of novel azaphenothiazine derivatives featuring tri-, tetra- and pentacyclic systems [3,4,5]. Structural modifications of phenothiazine and azaphenothiazine have been achieved by introducing substituents and functional moieties mainly at the thiazine nitrogen or, less often, into the benzene ring or nitrogen-containing heterocycles. The compounds reported so far exhibit neuroleptic, antimalarial, immunopotentiating, antibacterial, antiviral, antifungal, antiproliferative, or antitumor activities and can mediate the reversal of multidrug resistance [6,7,8,9,10,11,12,13]. In our earlier papers we reported a novel method of synthesizing the 1,4-thiazine ring which proceeds via the substitution of a hydrogen atom with a thiolate sulfur atom. The obtained compounds have demonstrated promising anticancer properties [14,15,16,17,18]. Herein, we report on the synthesis of novel tetracyclic phenothiazine derivatives containing various substituents (aliphatic, aromatic, or heterocyclic) in positions 9, 10, and 11 of the quinobenzothiazine system. Using selected neoplastic cell lines, we demonstrate the anticancer activity of these compounds and present results shedding light on their underlying mechanism of action.

## 2. Results and Discussion

### 2.1. Chemistry

**Scheme 1:**
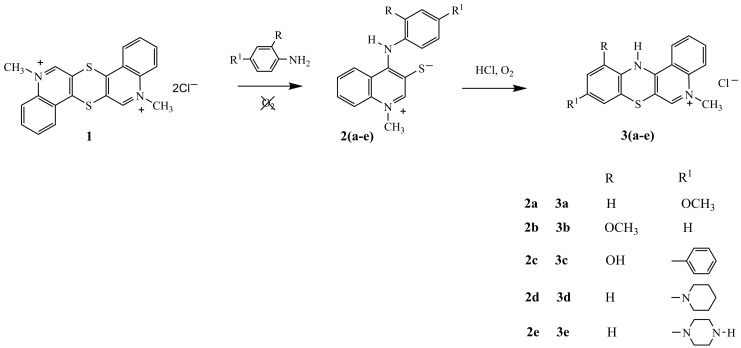
Synthesis of compounds **2a**–**e** and **3a**–**e**.

Cyclization of diphenylamines and azinylphenylamines in the presence of sulfur and its compounds, as well as cyclization of diphenyl or azinylphenyl sulfides are commonly used methods for the synthesis of phenothiazine and azaphenothiazine systems [2]. Our method of obtaining quinobenzothiazine tetracyclic derivatives is based on cyclization of betaine systems featuring 1-alkyl-4-(arylamino)quinolinium-3-thiolates structure via nucleophilic substitution of the hydrogen or halogen atom in the phenyl ring by a thiolate-derived sulfur atom. Reactions occur with high yield even at room temperature. They permit modification of the quinothiazine system by introducing substituents or functional groups into the benzene ring; this remains difficult when using other methods of synthesis [14,15,16]. Substrates which are suitable for obtaining 1-alkyl-4-arylaminoquinoline-3-thiolates are 5,12-(dialkyl)thioquinantrene salts **1** [19] as well as 1-alkyl-4-arylamino-3-(acylthio)quinolinium salts [20]. In this report we show synthesis of azaphenothiazine derivatives containing different types of aliphatic, aromatic and heterocyclic substituents in positions 9, 10 and 11 of the quinobenzothiazine system. The course of reaction between bis-chloride **1** with 2-, 3-, or 4-methoxyaniline, 4-piperidinylaniline, 4-piperazinylaniline, and 2-hydroxy-4-phenylaniline (Scheme 1) is dependent on the presence of oxygen in the reaction mixture.

When carried out at room temperature with pyridine as a solvent and under conditions disallowing oxygen access to the reaction milieu. These reactions led to 1-methyl-4-(arylamine)quinolinium-3-thiolates **2a**–**f**. Betaines **2a**–**e** underwent cyclization in the presence of a hydrogen chloride donor and atmospheric oxygen to appropriate quinobenzothiazine chlorides **3a**–**e** (procedure A). Compounds **3a**–**e** can also be obtained directly from bis-chloride **1** using suitable amines and a carrying one-pot type reaction in the presence of atmospheric oxygen and without separating intermediate products **2a**–**e** (procedure B).

**Scheme 2:**
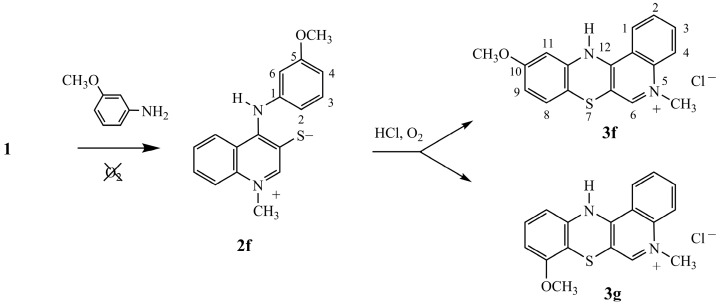
Cyclization reaction of quinolinium-3-thiolate **2f**.

The reaction of 1,4-thiazine ring formation during cyclization of quinolinium-3-thiolate **2f** obtained by reacting bis salt **1** with 3-methoxyaniline can occur via hydrogen atom substitution in positions 2 or 6 of the phenyl ring. ^1^H-NMR analysis has demonstrated the reaction product to be the mixture of isomers **3f** and **3g** (which form at a ca. 1:1 quantitative ratio) resulting from hydrogen atom substitution in both positions of the phenyl ring (Scheme 2). Compounds **3f** and **3g** obtained using the above method cannot be separated to purity.

**Scheme 3:**
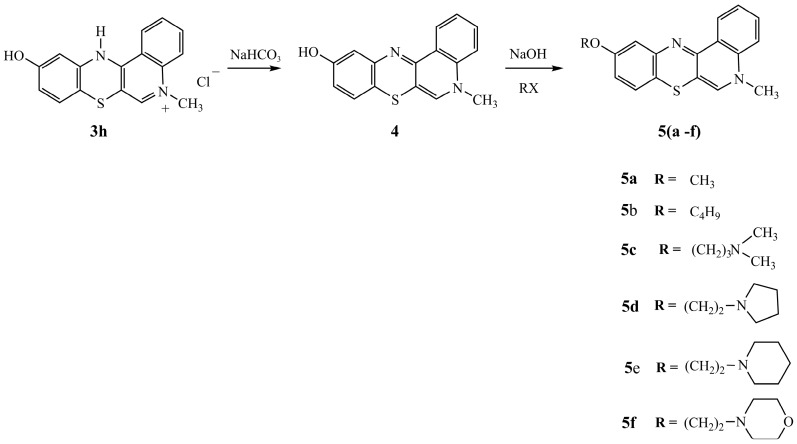
Synthesis of compounds **5a**–**f**.

In order to obtain quinobenzothiazine derivatives containing alkoxy and aminoalkoxy substituents in the position 10 of the quinobenzothiazine ring, we used 10-hydroxy-5-methyl-12(*H*)-quino[3,4-*b*][1,4]benzothiazinium chloride **3h**. Its synthesis was reported earlier [16]. Alkalization of the aqueous solution of compound **3h** with 5% NaHCO_3_ solution led to the elimination of hydrogen chloride and formation of quinobenzothiazine derivative **4** with quantitative yield. Alkylation of compound **4** in anhydrous 1,4-dioxane and in the presence of sodium hydroxide led to the corresponding 10-*O*-substituted quinobenzothiazine derivatives **5a**–**f** (Scheme 3).

**Scheme 4:**
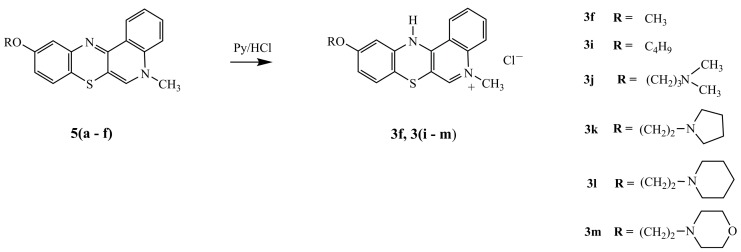
Synthesis of compounds **3f** and **3i**–**m**.

The present study was aimed at obtaining novel quinobenzothiazine derivatives that would contain various types of substituents in the phenyl ring of the quinobenzothiazine system and at examining their biological properties. Compounds **5a**–**f** are totally insoluble in water; this difficulty was omitted by transforming them into corresponding hydrochlorides **3f**, **3i**–**m** which are very soluble in water (Scheme 4).

In the experimental part we have provided ^1^H-NMR data for all newly obtained compounds. A number of them are not very soluble in solvents commonly used in NMR. For the chosen derivatives from each group of compounds (obtained via different reaction paths) we provide a full assignment of ^13^C-NMR signals (based on two-dimensional spectra analysis) which fully corroborate their structures.

### 2.2. Biological Activity and Antineoplastic Properties

The anticancer effect of neuroleptic phenothiazines containing alkylaminoalkyl substituents at the thiazine nitrogen atom was observed early. Novel phenothiazine derivatives and their antiproliferative effects and mechanisms have been the subject of numerous reports. Their results suggest that antiproliferative action results from an interaction between such derivatives and proteins involved in inducing apoptosis, as well as from DNA intercalating properties inducing its fragmentation in cancer cells. The examined quinobenzothiazine salts have planar structural fragments which may facilitate intercalation into the DNA helix, similar to that exhibited by anthracycline antibiotics. The presence of an intercalating factor (substituent or functional group capable of forming hydrogen bonds with purine and pyrimidine bases) increases stability of the DNA-drug complex and may result in inhibited cell proliferation. Synthesis of novel phenothiazine derivatives with anticancer properties was, until now, accomplished via modification of the basic structural fragment, mainly by introducing substituents at the thiazine nitrogen atom. In the case of quinobenzothiazine derivatives described earlier, the greatest antiproliferative activity was demonstrated by compounds with an amine group in the benzene ring [16]. In this report, we describe novel quinobenzothiazine derivatives containing various types of substituents in positions 9, 10, and 11 including heterocyclic amine systems at different distances from the quinobenzothiazine system. It could be expected that the presence of additional amine basic centers might augment antiproliferative activity of the investigated compounds due to the stabilization of compound-DNA complexes following formation of additional hydrogen bonds with the DNA helix. The synthesized compounds were tested using three cancer cell lines: MDA-MB-231 (breast adenocarcinoma), SNB-19 (glioblastoma), and C-32 (amelanotic melanoma).

#### 2.2.1. Cell Viability Studies

**Figure 1:**
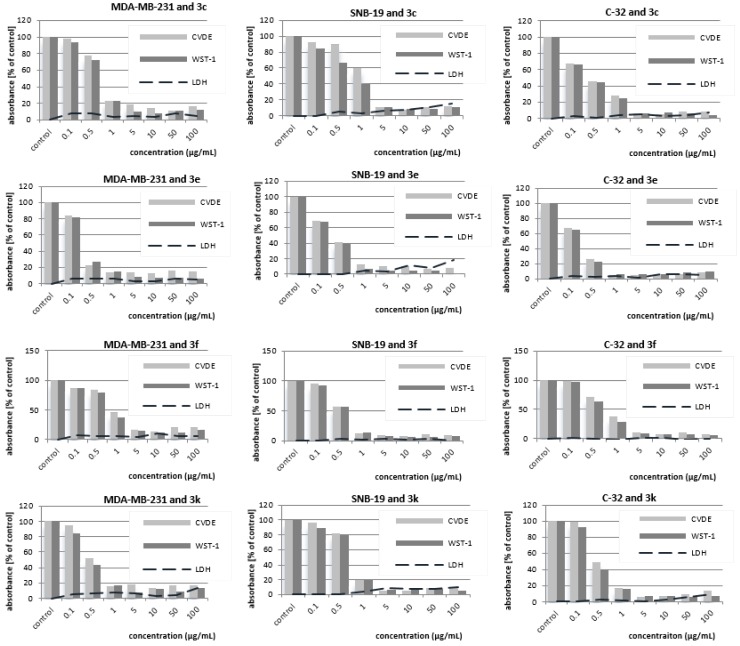
The effect of derivatives **3c**, **3e**, **3f**, and **3k** upon MDA-MB-231, SNB-19, and C-32 cancer cell lines (CVDE—cell number, WST-1—metabolic activity, LDH—number of dead cells in culture).

To assess the effect of the synthesized compounds on viability of cultured cells (dependent on cell number and metabolic activity), a WST-1 (a Water Soluble Tetrazolium salt) test was used. IC_50_ values for cell cultures exposed to the examined compounds for 72 h are shown in Table 1. All of the analyzed compounds inhibit growth of cultured cells (IC_50_ range 0.5–24.5 μM) when compared to control. The weakest antiproliferative activity was shown by compounds devoid of substituents with additional nitrogen atoms. Compounds **3d** and **3e** were the most active; they contain, respectively, piperidinyl and piperazinyl substituents linked directly to the ring in position 9. These are followed by derivatives **3k**, **3l**, and **3m**, featuring alkoxy substituents with nitrogen-containing heterocyclic rings (pyrrolidine, piperidine, and morpholine) in position 10. No significant effect towards the tested cell lines was observed in terms of the distance between amine basic centers and the quinobenzothiazine system Four of the compounds (**3c**, **3e**, **3f**, and **3k**) featuring different types of substituents were examined in more detail with respect to their effect on cultured cell lines; this was based on the assessment of crystal violet binding to DNA (CVDE, Crystal Violet Dye Elution test) as well as on the quantitation of dead cells based on mitochondrial dehydrogenase released into the culture medium (lactate dehydrogenase test—LDH). The four examined compounds showed activity on all three cancer cell lines studied. The derivatives substantially lower the number of cells in the culture (as compared to control); the effect is seen at 0.5 μg/mL and higher (Figure 1). This was not accompanied, however, by elevated lactate dehydrogenase levels which would otherwise point to high cytotoxicity of the examined compounds towards the type of cultured cells examined. The results of WST-1, CVDE, and LDH tests suggest, on the other hand, a blockade to cell division.

#### 2.2.2. Effect of Compounds on the Transcriptional Activity of H3, BCL-2, BAX, and TP53

**Figure 2:**
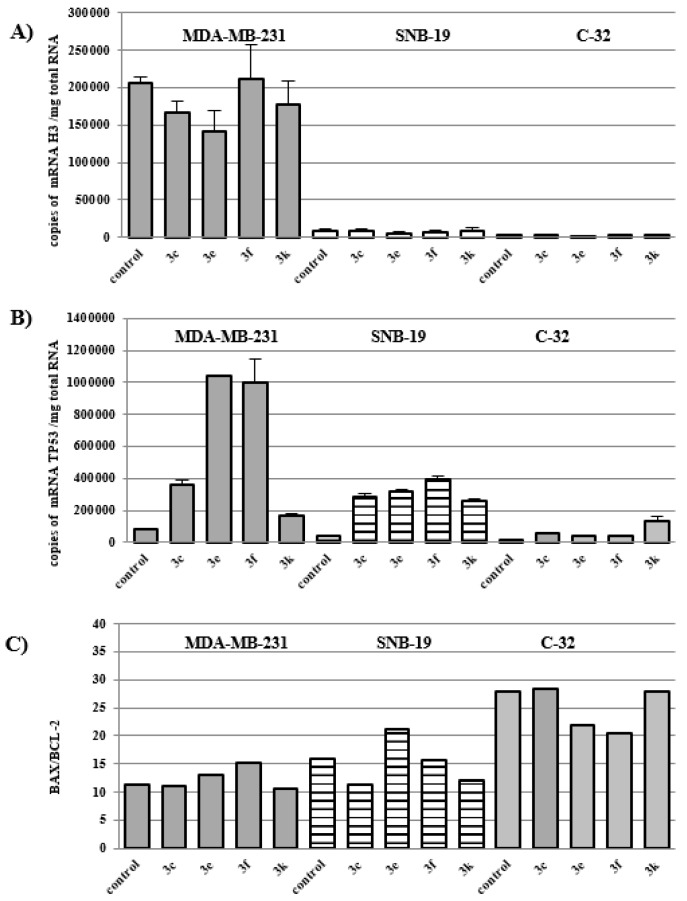
Effect of **3c**, **3e**, **3f**, and **3k** on transcriptional activity of: H3 (**A**); TP53 (**B**); BAX and BCL-2 (**C**) in MDA-MB-231, SNB-19, and C-32 cells.

We examined the effect of the tested compounds upon transcriptional activity of genes encoding a proliferation marker (H3 histone), two mitochondrial apoptosis pathway-involved proteins (BCL-2 and BAX), and a cell cycle regulator (p53) following a 24 h exposure of cultured cells to the tested compounds (0.5 µg/mL). H3 histone gene expression analysis corroborated the observed effect of a numerical decrease of cultured cells following the inhibition of proliferation. The results point to limited proliferative activity of cells under the experimental conditions tested (see H3 histone mRNA in Figure 2).

Increased copy numbers of mRNA encoding P53 protein suggest a commitment of exposed cells to stepped-up regulatory processes. The ratio of mRNA copies (proapoptotic BAX)to antiapoptotic BCL-2 which was maintained at constant levels, suggests that, despite elevated expression of TP53, the loss in cell number observed in cultures exposed to the tested compounds does not result from apoptosis but is caused by some other process (e.g., inability of cells to proliferate).

#### 2.2.3. DNA Binding of the Examined Compounds

**Figure 3:**
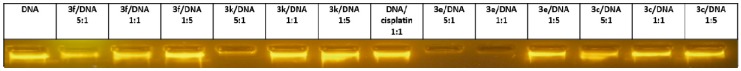
DNA intercalation with EtBr: 1 h incubation with derivatives **3c**, **3e**, **3f**, and **3k** (5/1, 1/1 and 1/5 *w*/*w*) (µg derivative/µg DNA).

Ethidium bromide (EtBr) is a DNA intercalator. Upon UV exposure of DNA-bound EtBr, its fluorescence increases. Incubation (1 h) of the selected novel derivatives with genomic DNA (at 5/1, 1/1, and 1/5 *w*/*w*) followed by the subsequent addition of EtBr, showed that the two derivatives **3e** (two highest concentrations: **3e**/DNA 5/1, 1/1 *w/w*) and **3k** (highest concentration: **3k**/DNA 5/1, *w*/*w*) can bind to DNA in amounts making DNA intercalation with EtBr impossible (Figure 3). The effect observed for **3e** and DNA at 1/1 compound/DNA weight ratio does not occur in the case of cisplatin and DNA.

## 3. Materials and Methods

### 3.1. Chemistry

Melting points are uncorrected. NMR spectra were recorded using a Bruker Ascend 600 spectrometer (Bruker, Billerica, MS, USA). To assign the structures, the following 2D experiments were employed: ^1^H-^13^C gradient selected HSQC (Heteronuclear Single Quantum Coherence) and HMBC (Heteronuclear Multiple Bond Coherence) sequences. Standard experimental conditions and standard Bruker program were used. The ^1^H- and ^13^C-NMR spectral data are given relative to the TMS signal at 0.0 ppm. EI MS spectra were recorded using an LKB GC MS 20091 spectrometer at 75 eV (LKB, Bromma, Sweden).

#### 3.1.1. Synthesis of 1-Methyl-4-(arylamino)quinolinium-3-thiolates **2**

Argon was passed through the suspension of bis-chloride (**1**) (0.419 g, 1 mmol) in dry pyridine (15 mL) at room temperature over 15 min. Amine (2.5 mmol) was added and the reaction mixture was bubbled with argon for a further 15 min. The mixture was then stirred at room temperature for 7 days. The solid product was filtered off and washed with dry ether. The raw product was purified through recrystallization from ethanol.

*1-Methyl-4-((4-methoxy)phenylamino)quinolinium-3-thiolate* (**2a**). Yield: 54%; m.p. 173 °C; ^1^H-NMR (DMSO_d-6_, 600 MHz) δ (ppm): 3.77 (s, 3H, OCH_3_), 4.16 (s, 3H, NCH_3_), 6.89–6.98 (m, 2H, H_arom_), 7.06–7.13 (m, 2H, H_arom_), 7.20–7.28 (m, 1H, H6_quinolinyl_), 7.42–7.48 (m, 1H, H8_quinolinyl_), 7.55–7.62 (m, 1H, H7_quinolinyl_), 7.88–7.94 (m, 1H, H5_quinolinyl_), 8.70 (s, 1H, H2_quinolinyl_), 9.99 (s, 1H, NH); ^13^C-NMR (DMSO_d-6_, 150.9 MHz) δ (ppm): 42.26 (NCH_3_), 55.79 (OCH_3_), 115.20 (C2′, C6′), 118.90 (C5), 124.54 (C4a), 124.65 (C8), 124.90 (C6), 124.99 (C3′, C5′), 129.57 (C7), 134.71 (C3), 135.74 (C8a), 145.00 (C2), 151.16 (C4′), 153.43 (C4), 157.47 (C1′); EI-MS (70eV) (*m/z*): 296 (M^+^, 100%); Anal. calcd. for C_17_H_16_N_2_OS: C, 68.89; H, 5.44; N, 9.45; S, 10.82. Found: C, 68.81; H, 5.35; N, 9.40; S, 8.78.

*1-Methyl-4-((2-methoxy)phenylamino)quinolinium-3-thiolate* (**2b**). Yield: 58%; m.p. 172–174 °C; ^1^H-NMR (DMSO_d-6_, 600 MHz) δ (ppm): 3.77 (s, 3H, OCH_3_), 4.19 (s, 3H, NCH_3_), 6.84–6.93 (m, 2H, H_arom_), 7.12–7.22 (m, 2H, H_arom_), 7.25–7.31 (m, 1H, H6_quinolinyl_), 7.45–7.49 (m, 1H, H8_quinolinyl_), 7.57–7.63 (m, 1H, H7_quinolinyl_), 7.89–7.94 (m, 1H, H5_quinolinyl_), 8.73 (s, 1H, H2_quinolinyl_), 9.86 (s, 1H, NH); EI-MS (70 eV) (*m/z*): 296 (M^+^, 100%); Anal. calcd. for C_17_H_16_N_2_OS: C, 68.89; H, 5.44; N, 9.45; S, 10.82. Found: C, 8.78; H, 5.39; N, 9.38; S, 8.75.

*1-Methyl-4-((2-hydroxy-4-phenyl)phenylamino)quinolinium-3-thiolate* (**2c**). Yield: 37%; m.p. 198 °C; ^1^H-NMR (DMSO_d-6_, 600 MHz) δ (ppm): 4.19 (s, 3H, NCH_3_), 7.03–7.08 (m, 1H, H_arom_), 7.15–7.18 (m, 1H, H_arom_), 7.22–7.26 (m, 1H, H_arom_), 7.28–7.37 (m, 3H, H_arom_), 7.37–7.40 (m, 1H, H_arom_), 7.40–7.48 (m, 2H, H_arom_), 7.60–7.67 (m, 1H, H7_quinolinyl_), 7.67–7.73 (m, 1H, H8_quinolinyl_), 7.93–7.98 (m, 1H, H5_quinolinyl_), 8.74 (s, 1H, H2_quinolinyl_), 9.97 (s, 1H, NH), 10.17 (s, 1H, OH), EI-MS (70 eV) (*m/z*): 358 (M^+^, 100%); Anal. calcd. for C_22_H_18_N_2_OS: C, 73.72; H, 5.06; N, 7.81; S, 8.94. Found: C, 73.67; H, 4.97; N, 7.76; S 8.91.

*1-Methyl-4-(4-(N-piperidinyl)phenylamino)quinolinium-3-thiolate* (**2d**). Yield: 84%; m.p. 208 °C; ^1^H-NMR (DMSO_d-6_, 600 MHz) δ (ppm): 1.45–1.80 (m, 6H, H_piperidinyl_), 3.10–3.30 (m, 4H, H_piperidinyl_), 4.15 (s, 3H NCH_3_), 7.03–7.08 (m, 4H, H_arom_), 7.25–7.38 (m, 1H, H_arom_), 7.55–7.74 (m, 1H, H_arom_), 7.92–7.98 (m, 1H, H_arom_), 8.69 (s, 1H, H2_quinolinyl_), 10.08 (s, 1H, NH); EI-MS (70 eV) (*m/z*): 349 (M^+^, 100%); Anal. calcd. for C_21_H_23_N_3_S: C, 72.17; H, 6.63; N, 12.02; S, 9.17. Found: C, 72.15; H, 6.54; N, 11.95; S, 9.13.

*1-Methyl-4-(4-(N-piperazinyl)phenylamino)quinolinium-3-thiolate* (**2e**). Yield: 86%; m.p. 137 °C; ^1^H-NMR (DMSO_d-6_, 600 MHz) δ (ppm): 3.05–3.35 (m, 8H, H_piperazinyl_), 4.17 (s, 3H, NCH_3_), 4.69 (s, 1H, NH_piperazinyl_), 6.96–7.02 (m, 2H, H_arom_), 7.06–7.10 (m, 2H, H_arom_), 7.25–7.30 (m, 1H, H_arom_), 7.51–7.55 (m, 1H, H_arom_), 7.58–7.64 (m, 1H, H_arom_), 7.90–7.96 (m, 1H, H_arom_), 8.70 (s, 1H, H2_quinolinyl_), 10.01 (s, 1H, NH); EI-MS (70 eV) (*m/z*): 350 (M^+^, 100%); Anal. calcd. for C_20_H_22_N_4_S: C, 68.54; H, 6.33; N, 15.99; S, 9.15. Found: C, 68.49; H, 6.25; N, 15.90; S, 9.10.

*1-Methyl-4-(3-methoxyphenylamino)quinolinium-3-thiolate* (**2f**). Yield: 66%; m.p. 181 °C; ^1^H-NMR (DMSO_d-6_, 600 MHz) δ (ppm): 3.69 (s, 3H, OCH_3_), 4.20 (s, 3H, NCH_3_), 6.58–6.63 (m, 1H, H_arom_), 6.69–6.77 (m, 1H, H_arom_), 7.20–7.26 (m, 1H, H4_arom_), 7.29–7.35 (m, 1H, H6_quinolinyl_), 7.49–7.54 (m, 1H, H8_quinolinyl_), 7.58–7.64 (m, 1H, H7_quinolinyl_), 7.94–7.99 (m, 1H, H5_quinolinyl_), 8.80 (s, 1H, H2_quinolinyl_), 9.91 (s, 1H, NH); EI-MS (70 eV) (*m/z*): 296 (M^+^, 100%); Anal. calcd for C_17_H_16_N_2_OS: C 68.89; H 5.44; N 9.45; S 10.82. Found: C, 68.79; H, 5.36; N, 9.40; S, 10.75.

#### 3.1.2. Synthesis of 5-Methyl-12(*H*)-quino[3,4-*b*][1,4]benzothiazinium Chloride **3**

Procedure (A): Aniline hydrochloride (0.155 g, 1.2 mmol) was added to the mixture of 3-thiolate (**2**) (1 mmol) in 10 mL of dry pyridine and the whole was mixed at 70 °C for 12 h. After cooling it down to room temperature, the formed precipitate was filtered off and washed with ether. The raw product was recrystallized from ethanol.

Procedure (B): Amine (2.5 mmol) was added to the mixture of bis-chloride (**1**) (0.419 g, 1 mmol) in 10 mL of dry pyridine and the whole was mixed at 70 °C for 12 h. The mixture was cooled down to room temperature and the formed precipitate was filtered off and washed with ether. The raw product was purified through recrystallization from ethanol.

*9-Methoxy-5-methyl-12(H)-quino[3,4-b][1,4]benzothiazinium chloride* (**3a**). Yield: Procedure (A) 68%, Procedure (B) 72%; ^1^H-NMR (DMSO_d-6_, 600 MHz) δ (ppm): 3.73 (s, 3H, OCH_3_), 4.19 (s, 3H, NCH_3_), 6.70–6.78 (m, 2H, H10, H11), 7.36–7.44 (m, 1H, H8), 7.76–7.84 (m, 1H, H2), 7.97–8.08 (m, 2H, H3, H4), 8.50–8.63 (m, 1H, H1), 8.84 (s, 1H, H6), 10.93 (s, 1H, NH); Anal. calcd. for C_17_H_15_ClN_2_OS: C, 61.72; H, 4.57; N, 8.47; S, 9.69. Found C, 61.63; H, 4.51; N, 8.41; S, 9.66.

*11-Methoxy-5-methyl-12(H)-quino[3,4-b][1,4]benzothiazinium chloride* (**3b**). Yield: Procedure (A) 65%, Procedure (B) 74%; ^1^H-NMR (DMSO_d-6_, 600 MHz) δ (ppm): 3.93 (s, 3H, OCH_3_), 4.18 (s, 3H, NCH_3_), 6.68–6.77 (d, ^3^*J* = 9 Hz, 1H, H8), 6.96–7.04 (d, ^3^*J* = 7.8 Hz, H10), 7.08–7.16 (d.d, ^3^*J* = 9 Hz, ^3^*J* = 7.8 Hz, H9), 7.82–7.90 (s, 1H, H2). 8.03–8.16 (m, 2H, H3, H4), 8.50–8.58 (m, 1H, H1), 8.80 (s, 1H, H6), 9.90 (s, 1H, NH); ^13^C-NMR (DMSO_d-6_, 150.9 MHz) δ (ppm): 43.28 (NCH_3_), 56.94 (OCH_3_), 107.54 (C6a), 112.28 (C12b), 116.28 (C8), 118.83 (C12a), 119.28 (C11), 119.32 (C10), 123.88 (C2), 125.40 (C11a), 128.10 (C9), 128.47 (C1), 134.99 (C3), 139.11 (C6), 144,20 (C4a), 148.90 (C7a), 152.03 (C4); Anal. calcd. for C_17_H_15_ClN_2_OS: C, 61.72; H, 4.57; N, 8.47, S, 9.69. Found: C, 6.66; H, 4.49; N, 8.45; S, 9.64.

*11-Hydroxy-9-phenyl-5-methyl-12(H)-quino[3,4-b][1,4]benzothiazinium chloride* (**3c**). Yield: Procedure (A) 59%, Procedure (B) 65%; ^1^H-NMR (DMSO_d-6_, 600 MHz) δ (ppm): 4.06 (s, 3H, NCH_3_), 6.93–7.02 (m, 2H, H_arom_), 7.30–7.35 (m, 2H, H_arom_), 7.38–7.44 (m, 1H, H_arom_), 7.44–7.51 (m, 2H, H_arom_), 7.74–7.85 (m, 1H, H_arom_), 7.98–8.07 (m, 2H, H_arom_), 8.60–8.65 (m, 2H, H_arom_), 10.10 (s, 1H, NH), 11.18 (s, 1H, OH); Anal. calcd. for C_22_H_17_ClN_2_OS: C, 67.25; H, 4.36; N, 7.13; S, 8.16. Found: C, 67.18; H, 4.30; N, 7.04; S 8.10.

*9-(N-piperidinyl)-5-methyl-12(H)-quino[3,4-b][1,4]benzothiazinium chloride* (**3d**). Yield: Procedure (A) 68%, Procedure (B) 77%; ^1^H-NMR (DMSO_d-6_, 600 MHz) δ (ppm): 1.43–1.70 (m, 6H, H_piperidinyl_), 3.08–3.20 (m, 4H, H_piperidinyl_). 4.05 (s, 3H, NCH_3_), 6.56–6.62 (d, ^4^*J* = 2.4 Hz, 1H, H8), 6.64–6.69 (d.d, ^3^*J* = 9 Hz, ^4^*J* = 2.4 Hz, 1H, H10), 7.44–7.50 (d, ^3^*J* = 9 Hz, 1H, H11), 6.70–6.75 (m, 1H, H2), 7.74–7.79 (m, 2H, H3, H4), 8.50 (s, 1H, H6), 9.01–9.06 (m, 1H, H1), 11.22 (s, 1H, NH). Anal. calcd. for C_21_H_22_ClN_3_S: C, 65.70; H, 5.78; N, 10.94; S, 8.35. Found C, 65.64; H, 5.73; N, 10.89; S, 8.32.

*9-(N-piperazinyl)-5-methyl-12(H)-quino[3,4-b][1,4]benzothiazinium chloride* (**3e**). Yield: Procedure (A) 66%, Procedure (B) 74%; ^1^H-NMR (DMSO_d-6_, 600 MHz) δ (ppm): 3.28–3.32 (m, 4H, H_piperazinyl_), 3.34–3.39 (m, 4H, H_piperazinyl_), 4.11 (s, 3H, NCH_3_) 6.65–6.69 (d, ^4^*J* = 3 Hz, 1H, H8), 6.72–6.77 (d, ^3^*J* = 9 Hz, 1H, H11), 6.77–6.82 (d.d, ^3^*J* = 9 Hz, ^4^*J* = 3 Hz, 1H, H10), 7.75–7.83 (m, 1H, H_arom_), 7.97–8.04 (m, 2H, H_arom_), 8.26 (s, 1H, H6), 8.48–8.53 (m, 1H, H_arom_); Anal. calcd. for C_20_H_21_ClN_4_S: C, 62.41; H, 5.50; N, 14.56; S, 8.33. Found C, 62.32; H, 5.44; N, 14.47; S, 8.28.

#### 3.1.3. Synthesis of 10-Hydroxy-5-methyl-5(*H*)-quino[3,4-*b*][1,4]benzothiazine **4**

Quinobenzothiazinium chloride (**3h**) (0.317 g, 1 mmol) was dissolved in 20 mL water (50 °C), the resulting solution was filtered and alkalized while mixing by using a 5% aqueous NaHCO_3_ solution (10 mL). The obtained solid product was filtered off and air-dried. Finally, the crude product was purified by recrystallization from ethanol.

*10-Hydroxy-5-methyl-5(H)-quino[3,4-b][1,4]benzothiazine* (**4**). Yield: 100%; m.p. 107–110 °C; ^1^H-NMR (CD_3_OD_d-4_, 600 MHz) δ (ppm): 3.90 (s, 3H, CH_3_), 6.42–6.47 (d.d, ^3^*J* = 8.4 Hz, ^4^*J* = 2.4 Hz, 1H, H9), 6.58 (s, 1H, H6), 6.61–6.67 (m, 1H, H_arom_), 7.53–7.61 (m, 1H, H_arom_), 7.66–7.74 (m, 2H, H_arom_), 7.78–7.86 (m, 1H, H_arom_), 8.34–8.39 (m, 1H, H_arom_); EI-MS (70 eV) (*m/z*): 280 (M^+^, 100%); Anal. calcd. for C_16_H_12_N_2_OS: C, 68.55; H, 4.31; N, 9.99; S, 11.44. Found: C, 68.53; H, 4.26; N, 9.94; S, 11.42.

#### 3.1.4. Synthesis of 10-Alkoxy-5-methyl-5(*H*)-quino[3,4-*b*][1,4]benzothiazine **5**

Anhydrous 1,4-dioxane (15 mL) was mixed with quinobenzothiazine (**4**) (0.28 g, 1 mmol) and sodium hydroxide (0.2 g, 5 mmol) and refluxed with mixing for 2 h. An alkylating agent (alkyl iodides or aminoalkyl chlorides) (1.3 mmol) was added stepwise and the mixture was refluxed for a subsequent 2 h. After cooling down to room temperature, the reaction mixture was poured into 50 mL of water and extracted with 15 mL chloroform. The resulting solution was dried over anhydrous calcium chloride and evaporated under vacuum. The dry residue was purified by chromatography using a silica gel-filled column and chloroform-ethanol (10:1 *v*/*v*) as eluent.

*10-Methoxy-5-methyl-5(H)-quino[3,4-b][1,4]benzothiazine* (**5a**). Yield: 30%; m.p. 268–270 °C; ^1^H-NMR (DMSO_d-6_, 600 MHz) δ (ppm): 3.51 (s, 3H, NCH_3_), 3.68 (s, 3H, OCH_3_), 6.38–6.44 (m, 2H, H_arom_), 6.56–6.60 (m, 1H, H_arom_), 7.07 (s, 1H, H6), 7.21–7.26 (m, 1H, H_arom_), 7.27–7.30 (m, 1H, H_arom_), 7.52–7.57 (m, 1H, H_arom_), 8.18–8.22 (m, 1H, H_arom_); ^13^C-NMR (DMSO_d-6_, 150.9 MHz) δ (ppm): 40.51 (NCH_3_), 55.46 (OCH_3_), 103.64 (12b), 111.09 (7a), 111.33(C9 or C11), 112.53 (C9 or C11), 115.89 (C4), 121.29 (6a), 123.96 (C2), 125.02 (C1), 126.01 (C8), 131.98 (3), 133.14 (C6), 140.75 (4a), 146.30 (C11a), 154.13 (C12a), 159.46 (C10); EI-MS (70 eV) (*m/z*): 294 (M^+^, 100%); Anal. calcd. for C_17_H_14_N_2_OS: C, 69.36; H, 4.79; N, 9.52; S, 10.89. Found: C, 69.29; H, 4.75; N, 9.48; S, 10.84.

*10-Butyloxy-5-methyl-5(H)-quino[3,4-b][1,4]benzothiazine* (**5b**). Yield: 37%; m.p. 280–282 °C; ^1^H-NMR (CDCl_3_, 600 MHz) δ (ppm): 2.58 (m, 3H, CH_3_), 2.70–2.80 (m, 2H, CH_2_), 3.38–3.50 (m, 2H, CH_2_), 3.76 (s, 3H, NCH_3_), 4.03–4.11 (t, *J* = 4.8 Hz, 2H, OCH_2_), 6.31–6.45 (m, 2H, H_arom_), 6.52–6.54 (m, 1H, H_arom_), 6.54–6.63 (m, 1H, H_arom_), 6.95–7.03 (m, 1H, H_arom_), 7.18–7.25 (m, 1H, H_arom_), 7.40–7.50 (m, 1H, H_arom_), 8.30–8.45 (m, 1H, H_arom_); EI-MS (70 eV) (*m/z*): 336 (M^+^, 100%); Anal. calcd. for C_20_H_20_N_2_OS: C, 71.40; H, 5.99; N, 8.33; S, 9.53. Found: C, 71.30; H, 5.94; N, 8.28; S, 9.49.

*10-(3-(N,N-dimethylamino)propyl)oxy-5-methyl-5(H)-quino[3,4-b][1,4]benzothiazine* (**5c**). Yield: 49%; m.p. 82–83 °C; ^1^H-NMR (DMSO_d-6_, 600 MHz) δ (ppm): 2.10–2.20 (t, ^3^*J* = 6 Hz, 2H, NCH_2_), 2.76 (s, 3H, NCH_3_), 2.77 (s, 3H, NCH_3_), 3.11–3.25 (m, 2H, CH_2_), 3.95–4.05 (t, ^3^*J* = 6 Hz, 2H, OCH_2_), 4.15 (s, 3H, NCH_3_), 6.65–6.75 (d.d, ^3^*J* = 8.4 Hz, ^4^*J* = 2.4 Hz, 1H, H9), 6.92–7.00 (d, ^3^*J* = 8.4 Hz, 1H, H8), 7.45–7.50 (d, ^4^*J* = 2.4 Hz, 1H, H11), 7.75–7.84 (m, 1H, H_arom_), 7.95–8.12 (m, 2H, H_arom_), 8.69 (s, 1H, H6), 9.15–9.27 (m, 1H, H_arom_); EI-MS (70 eV) (*m/z*): 365 (M^+^, 100%); Anal. calcd. for C_21_H_23_N_3_OS: C, 69.01; H, 6.34; N, 11.50; S, 8.77. Found: C, 68.96; H, 6.30; N, 11.47; S, 8.75.

*10-(2-(N-pyrrolidinyl)ethyl)oxy-5-methyl-5(H)-quino[3,4-b][1,4]benzothiazine* (**5d**). Yield: 46%; m.p. 148–150 °C; ^1^H-NMR (DMSO_d-6_, 600 MHz) δ (ppm): 1.62–1.76 (m, 4H, H_pyrrolidinyl_), 2.52–2.57 (m, 4H, H_pyrrolidinyl_), 2.73 (t, *J* = 6 Hz, 2H, NCH_2_), 3.51 (s, 3H, NCH_3_), 3.98 (t, *J* = 6 Hz, 2H, OCH_2_), 6.37–6.44 (m, 2H, H_arom_), 6.55–6.59 (m, 1H, H_arom_), 7.07 (s, 1H, H6), 7.22–7.25 (m, 1H, H_arom_), 7.25–7.30 (m, 1H, H_arom_), 7.52–7.57 (m, 1H, H_arom_), 8.17–8.24 (m, 1H, H_arom_); EI-MS (70 eV) (*m/z*): 377 (M^+^, 73%); Anal. calcd. for: C_22_H_23_N_3_OS: C, 70.00; H, 6.14; N, 11.13; S, 8.49. Found: C, 69.96; H, 6.08; N, 11.09; S, 8.48.

*10-(2-(N-piperidinyl)ethyl)oxy-5-methyl-5(H)-quino[3,4-b][1,4]benzothiazine* (**5e**). Yield: 67%; m.p. 70–73 °C; ^1^H-NMR (DMSO_d-6_, 600 MHz) δ (ppm): 1.30–1.40 (m, 2H, H_piperidinyl_), 1.40–1.53 (m, 4H, H_piperidinyl_), 2.35–2.45 (m, 4H, H_piperidinyl_), 2.54–2.61 (t, *J* = 6 Hz, 2H, NCH_2_), 3.51 (s, 3H, NCH_3_), 3.93–4.01 (t, ^3^*J* = 6 Hz, 2H, OCH_2_), 6.35–6.40 (m, 2H, H_arom_), 6.52–6.59 (m, 1H, H_arom_), 7.06 (s, 1H, H6), 7.15–7.32 (m, 2H, H_arom_), 7.49–7.55 (m, 1H, H_arom_), 8.15–8.22 (m, 1H, H_arom_); EI-MS (70 eV) (*m/z*): 392 (M^+^, 100%); Anal. calcd. for C_23_H_25_N_3_OS: C, 70.56; H, 6.44; N, 10.73; S, 8.19. Found: C, 70.48; H, 6.49; N, 10.70; S, 8.17.

*10-(2-(N-morpholinyl)ethyl)oxy-5-methyl-5(H)-quino[3,4-b][1,4]benzothiazine* (**5f**). Yield: 15%; m.p. 196–198 °C; ^1^H-NMR (DMSO_d6_, 600 MHz) δ (ppm): 2.38–2.45 (m, 4H, H_morpholinyl_), 2.57–2.65 (t, *J* = 5.4 Hz, 2H, NCH_2_), 3.51 (s, 2H, NCH_3_), 3.53–3.62 (m, 4H, H_morpholinyl_), 3.92–4.05 (t, *J* = 5.4 Hz, 2H, OCH_2_), 6.38–6.44 (m, 2H, H_arom_), 6.55–6.59 (m, 1H, H_arom_), 7.07 (s, 1H, H6), 7.20–7.32 (m, 2H, H_arom_), 7.48–7.57 (m, 1H, H_arom_), 8.18–8.24 (m, 1H, H_arom_). EI-MS (70 eV) (*m/z*): 394 (M^+^, 100%); Anal. calcd. for C_22_H_23_N_3_O_2_S: C, 67.15; H, 5.89; N, 10.68; S, 8.15. Found: C, 67.10; H, 5.83; N, 10.62; S 8.12.

#### 3.1.5. Synthesis of 12(*H*)-quino[3,4-*b*][1,4]benzothiazinium Chloride **3f**, **3i**–**m**

Quinobenzothiazine (**5**) (1 mmol) was dissolved in 15 mL anhydrous ethanol. Pyridine hydrochloride (1 mmol) was added and the reaction mix was refluxed for 2 h. Solvents were evaporated under vacuum and the dried remaining residue was purified using a chromatography column filled with aluminum oxide and chloroform:ethanol (10:1 *v*/*v*) as eluent.

*10-Methoxy-5-methyl-12(H)-quino[3,4-b][1,4]benzothiazinium chloride* (**3f**).Yield: 87%; ^1^H-NMR (DMSO_d-6_, 600 MHz) δ (ppm): 3.75 (s, 3H, NCH_3_), 4.15 (s, 3H, OCH_3_), 6.68–6.72 (m, 1H, H_arom_), 6.98–7.02 (m, 1H, H_arom_), 7.24–7.26 (d, ^4^*J* = 3 Hz, H11), 7.83–7.88 (m, 1H, H_arom_), 8.01–8.12 (m, 2H, H_arom_), 8.68 (s, 1H, H6), 8.92–8.98 (m, 1H, H_arom_), 10.97 (s, 1H, NH). Anal. calcd. for C_17_H_15_ClN_2_OS: C, 61.72; H, 4.57; N, 8.47; S, 9.69. Found: C, 61.65; H, 4.51; N, 8.42; S, 9.66.

*10-Butyloxy-5-methyl-12(H)-quino[3,4-b][1,4]benzothiazinium chloride* (**3i**). Yield 82%; ^1^H-NMR (DMSO_d-6_, 600 MHZ) δ (ppm): 3.15–3.25 (m, 3H, CH_3_), 3.71–3.85 (m, 2H, CH_2_), 3.95–4.02 (m, 2H, CH_2_), 4.15 (s, 3H, NCH_3_), 4.34–4.43 (t, *J* = 4.2 Hz, 2H, OCH_2_), 6.75–6.80 (d.d, ^3^*J* = 8.4 Hz, ^4^*J* = 2.4 Hz, 1H, H9), 7.03–7.08 (d, ^3^*J* = 8.4 Hz, 1H, H8), 7.25–7.31 (d, ^4^*J* =2.4 Hz, 1H, H11), 7.82–7.89 (m, 1H, H_arom_), 8.04–8.12 (m, 2H, H_arom_), 8.69 (s, 1H, H6), 8.91–8.97 (m, 1H, H_arom_), 11.11 (s, 1H, NH). Anal. calcd. for C_20_H_21_ClN_2_OS: C, 64.42; H, 5.68; N, 7.51; S, 8.60. Found: C, 64.38; H, 5.63; N, 7.75; S 8.57.

*10-(3-(N,N-Dimethylamino)propyl)oxy-5-methyl-12(H)-quino[3,4-b][1,4]benzothiazinium chloride* (**3j**). Yield: 80%; ^1^H-NMR (DMSO_d-6_, 600 MHz) δ (ppm): 2.16 (t, *J* = 6 Hz, 2H, NCH_2_), 2.77 (s, 3H, NCH_3_), 2.78 (s, 3H, NCH_3_), 3.19 (m, 2H, CH_2_), 4.04 (t, *J* = 6 Hz, 2H, OCH_2_), 4.15 (s, 3H, NCH_3_), 6.67–6.73 (d.d, ^3^*J* = 8.4 Hz, ^4^*J* = 2.4 Hz, 1H, H9), 6.98–7.02 (d, ^3^*J* = 8.4 Hz, 1H, H8), 7.48–7.53 (d, ^4^*J* = 2.4 Hz, 1H, H11), 7.78–7.85 (m, 1H, H_arom_), 8.02–8.11 (m, 2H, H_arom_), 8.69 (s, 1H, H6), 9.20–9.25 (m, 1H, H_arom_), 10.73–10.80 (m, 1H, H_arom_), 11.39 (s, 1H, NH); ^13^C-NMR (DMSO_d-6_, 150.9 MHz) δ (ppm): 24.16 (NCH_2_), 42.49 (N(CH_3_)_2_), 43.11 (NCH_3_), 54.30 (CH_2_), 65.72 (OCH_2_), 106.67 (C6a), 106.96 (C11), 107.40 (7a), 113.22 (C9), 116.17 (11a), 119.16 (C4), 124.88 (C1), 127.92 (C8), 128.23 (C12a), 134.85 (C2), 138.05 (C3), 139.10 (C4a), 143.69 (C6), 151.87 C12a), 158.92 (C10); Anal. calcd. for C_21_H_24_ClN_3_OS: C, 62.75; H, 6.02; N, 10.45; S, 7.98. Found: C, 62.71; H, 5.96; N, 10.40; S, 7.94.

*10-(2-(N-Pyrrolidinyl)ethyl)oxy-5-methyl-12(H)-quino[3,4-b][1,4]benzothiazinium chloride* (**3k**). Yield: 83%; ^1^H-NMR (DMSO_d-6_, 600 MHz) δ (ppm): 1.80–1.90 (m, 2H, H_pyrrolidinyl_), 1.90–2.05 (m, 2H, H_pyrrolidinyl_), 3.02–3.12 (m, 2H, NCH_2_), 3.51–3.62 (m, 4H, H_pyrrolidinyl_), 4.11 (s, 3H, NCH_3_), 4.30–4.35 (t, *J* = 4.8 Hz, 2H, OCH_2_), 6.80–6.85 (m, 2H, H_arom_), 7.50–7.56 (m, 1H, H_arom_), 7.78–7.84 (m, 1H, H_arom_), 8.02–8.07 (m, 1H, H_arom_), 8.61 (s, 1H, H6), 8.95–9.00 (m, 1H, H_arom_), 10.95–11.04 (m, 1H, H_arom_), 11.13 (s, 1H, NH); Anal. calcd. for C_22_H_24_ClN_3_OS: C, 63.83; H, 5.84; N, 10.15; S, 7.74. Found: C, 63.86; H, 5.78; N, 10.11; S, 7.70.

*10-(2-(N-Piperidinyl)ethyl)oxy-5-methyl-12(H)-quino[3,4-b][1,4]benzothiazinium chloride* (**3l**). Yield: 86%; ^1^H-NMR (DMSO_d-6_, 600 MHz) δ (ppm): 1.32–1.45 (m, 2H, H_piperydyl_), 1.70–1.90 (m, 4H, H_piperydyl_), 2.95–3.10 (t, *J* = 4.8 Hz, 2H, NCH_2_), 3.45–3.50 (m, 4H, H_piperydyl_), 4.15 (s, 3H, NCH_3_), 4.38–4.43 (t, *J* = 4.8 Hz, 2H, OCH_2_), 6.73–6.78 (d.d, ^3^*J* = 9 Hz, ^4^*J* = 3 Hz, 1H, H9), 7.01–7.05 (d, ^3^*J* = 9 Hz, 1H, H8), 7.32–7.34 (d, ^3^*J* = 3 Hz, 1H, H11), 7.81–7.88 (m, 1H, H_arom_), 8.01–8.10 (m, 2H, H_arom_), 8.69 (s, 1H, H6), 8.98–9.04 (m, 1H, H_arom_), 10.46 (s, 1H, NH). Anal. calcd. for C_23_H_26_ClN_3_OS: C, 64.55; H, 6.12; N, 9.82; S, 7.49. Found: C, 64.50; H, 6.06; N, 9.78; S, 7.45.

*10-(2-(N-morpholinyl)ethyl)oxy-5-methyl-12(H)-quino[3,4-b][1,4]benzothiazinium chloride* (**3m**). Yield: 100%; ^1^H-NMR (DMSO_d-6_, 600 MHz) δ (ppm): 3.12–3.33 (m, 4H, H_morpholinyl_), 3.85–4.02 (m, 6H, NCH_2_, 4H_morpholinyl_), 4.16 (s, 3H, NCH_3_), 4.38–4.45 (t, *J* = 4.2 Hz, OCH_2_), 6.75–6.78 (d.d, ^3^*J* = 9 Hz, ^4^*J* = 2.4 Hz, H9), 7.02–7.06 (d, ^3^*J* = 9 Hz, 1H, H8), 7.48–7.53 (d, ^4^*J* = 2.4 Hz, 1H, H11), 7.78–7.68 (m, 1H, H_arom_), 8.03–8.12 (m, 2H, H_arom_), 8.71 (s, 1H, H6), 9.16–9.20 (m, 1H, H_arom_), 11.38 (s, 1H, NH). Anal. calcd. for C_22_H_24_ClN_3_OS: C, 61.46; H, 5.63; N, 9.77; S, 7.46. Found: C, 61.42; H, 5.59; N, 7.73; S, 7.44.

### 3.2. Biological Assays

#### 3.2.1. Cell Culture

Biological activity of the examined compounds was assessed in vitrousing three cultured cell lines: (1) MDA-MB-231 invasive breast ductal carcinoma (ATCC, Rockville, MD, USA); (2) SNB-19 glioblastoma (DSMZ, Braunschweig, Germany); (3) C-32 amelanotic melanoma (ATCC, Rockville, MD, USA). Cell cultures were maintained using DMEM (Lonza, Basel, Switzerland) supplemented with 10% fetal bovine serum (FBS) (Biological Industries Cromwell, CT, USA) and penicillin (10,000 U/mL)—streptomycin (10 mg/mL) mix (Lonza, Basel, Switzerland).

#### 3.2.2. Effect of Compounds on Number and Viability of Cells

Cells were cultured using 96 well plates (Nunc Thermo Fisher Scientific, Waltham, MA, USA). Cells were seeded (5 × 10^3^ cells/well) and incubated for 24 h in a standard incubator (37 °C, 5% CO_2_, relative humidity 95%). Next, medium was replaced with fresh aliquot containing an examined compound (0.1; 0.5; 1; 5; 10; 50; 100 µg/mL) and cells were further incubated for 72 h. Upon conclusion of incubation, a CVDE test (Aniara, West Chester, OH, USA) using crystal violet was performed to assess the relative number of cells in the culture. A WST-1 test (Roche) was performed to examine the metabolic activity of cells and test for the presence of LDH in the medium (Roche Diagnostics GmbH, Mannheim, Germany), allowing for the assessment of the relative number of dead cells (i.e., cytotoxicity of the examined compound). UVM340 microplate readers (BIOGENET, Józefów, Poland) were used to read absorbance values.

##### CVDE Test

AKCV96.1200 kit (Aniara) was used according to the manufacturer’s instructions. The test is based on cell membrane penetration by crystal violet and its ultimate interaction with DNA. Excess dye was washed off, cells were lysed, and absorbance measurements were performed for test and control samples at λ = 540 nm.

##### WST-1 Test

WST-1 colorimetric assay for cell proliferation (Roche Diagnostics GmbH, Mannheim, Germany, reagent kit cat. 11644807001) is based on the viable cells’ ability to cleave the bright red-colored stable tetrazolium salt WST-1 to dark red soluble formazan. This bioreduction occurs under the influence of mitochondrial dehydrogenases (depends mostly on production of NAD(P)H in viable cells). The amount of formazan dye formed correlates directly with the number of metabolically active cells in the culture and is measured by absorbance (λ = 450 nm) following 1 h incubation of cells with the reagent.

##### LDH Test

Lactate dehydrogenase (LDH) (Roche Diagnostics GmbH, Mannheim, Germany, reagent kit cat. 11644793001) is a cytosolic enzyme released into the culture medium following cell membrane damage. It can be used to assess the degree of toxicity of the examined substance. Relative number of dead cells (vs. control) in culture was determined using the LDH cytotoxicity detection kit (Roche Diagnostics GmbH, Mannheim Germany) according to the manufacturer’s protocol. Aliquots (100 μL) of the prepared reagent were added to media transferred (fresh plate) from wells with growing cells. Absorbance measurements (λ = 490 nm) of the samples were performed after 1 h.

#### 3.2.3. Transcriptional Activity of H3, BCL-2, BAX and TP53 Genes

Transcriptional activity of the following genes was assessed: *H3*/encoding histone H3, a proliferation marker/*BCL-2* and *BAX*/encoding BCL-2 and BAX, respectively, two apoptosis-related mitochondrial proteins/ and *TP53*/encoding P53, a cell cycle regulator/. The activity was assessed by RT-QPCR using Opticon™ DNA Engine system (MJ Research, Watertown, NY, USA) and QuantTect^®^ SYBR^®^ Green RT-PCR Kit (Qiagen, Hilden, Germany). Cultured cells were exposed (24 h) to the examined compounds (0.5 μg/mL). RNA was extracted using Quick-RNA™ MiniPrep kit columns (Zymo Research, Irvine, CA, USA). The extracted RNA was assessed qualitatively and quantitatively. Integrity of total RNA was checked by electrophoresis (1.2% agarose gel, EtBr). Amount and purity of the total RNA in extracts was determined spectrophotometrically (HP8452A apparatus, Hewlett Packard, Waldbronn, Germany).

#### 3.2.4. DNA Binding by the Examined Compounds

Genomic DNA was extracted from cells using silica bed columns (DNA isolation and purification kits). The obtained material was analyzed qualitatively and quantitatively by spectrophotometry (GeneQuant II analyzer, Pharmacia Biotech, Madrid, Spain). Extracted DNA samples were mixed either with an examined compound at 5:1, 1:1, 1:5 (*w*/*w*) ratios, or with cisplatin at 1:1 (*w*/*w*) ratio using 1–5 μg aliquots of the compound and DNA. Samples were applied to 0.9% agarose gel containing 0.5 mg/mL ethidium bromide (Promega, Fitchburg, WI, USA). The latter is a DNA intercalator the UV-induced fluorescence of which increases upon DNA binding. Cisplatin forms inter- and intrastrand crosslinks within DNA.

## 4. Conclusions

Reactions of tioquinantrenediinium bis-salts (**1**) with aromatic amines lead to tetracyclic quinobenzothiazine derivatives (**3**). Intermediate products of these reactions are 1-alkyl-4-aminoquinolinium-3-thiolates (**2**), the cyclization of which in the presence of atmospheric oxygen and a hydrogen chloride donor leads to the formation of 1,4-thiazine ring. Using this method of synthesis, novel derivatives were obtained containing hydroxyl, methoxyl, phenyl, piperidyl, and piperazinyl substituents in positions 9 and 11 of the quinobenzothiazine system. Alkylation of the hydroxyl group in position 10 allowed for the introduction of additional substituents such as alkyl, aminoalkyl, and aminoalkyl with heterocyclic nitrogen-containing rings (pyrrolidine, piperidine, and morpholine). Four compounds selected for further analysis were active towards the three tested cancer cell lines (MDA-MB-231, SNB-19, and C-32) (IC_50_ range 0.5–24.5 μM). Based on the results of CVDE, WST-1, and LDH tests it may be concluded that exposition to these compounds causes concentration-dependent reduction in the number of cultured cells. The four leading compounds can be ranked in the following order: **3e** > **3k** > **3f** > **3c**. The observed antiproliferative effect is not the result of the cytotoxic action of these derivatives leading to cell death; rather, it is the consequence of inhibited cell proliferation. The inhibition appears to be the result of these compounds binding to cellular DNA. The greatest inhibiting activity was demonstrated by compounds containing additional amine moieties in the structure. Their presence in the molecule could stabilize complex compound-DNA by enabling formation of additional hydrogen bonds with purine and pyrimidine bases in the DNA. The strongest DNA binding was observed for derivative (**3e**) containing a piperazine ring in position 9 and for derivative (**3k**) with a piperazine ring-containing substituent in position 10.

## Figures and Tables

**Table 1:** Effect of 5-methyl-12(*H*)-quino[3,4-*b*][1,4]benzothiazinium salts (**3**) and cisplatin (reference) on the viability of cells from three cancer cell lines studied.

Compound	IC_50_ (μM) ^a^		
	C-32	SNB-19	MDA-MB-231
**3a**	3.6 ± 0.9	3.9 ± 0.9	12.7 ± 1.5
**3b**	22.1 ± 2.4	22.7 ± 4.2	24.5 ± 3.0
**3c**	4.2 ± 1.0	11.5 ± 3.1	8.4 ± 2.3
**3d**	09 ± 0.5	1.0 ± 05	2.1 ± 0.5
**3e**	0.5 ± 0.3	0.8 ± 0.5	1.6 ± 0.5
**3f**	3.6 ± 1.2	2.7 ± 0.9	3.6 ± 1.2
**3i**	7.5 ± 1.6	6.7 ± 1.9	2.6 ± 0.5
**3j**	2.7 ± 1.5	2.2 ± 1.0	2.0 ± 0.7
**3k**	1.2 ± 0.7	0.7 ± 0.5	0.5 ± 0.2
**3l**	1.2 ± 0.7	1.2 ± 0.7	3.0 ± 0.9
**3m**	0.7 ± 0.2	1.8 ± 0.7	1.8 ± 0.5
cisplatin	11.0 ± 0.7	12.3 ± 1.0	25.0 ± 0.7

^a^ The results are from five independent experiments.
